# The Associations between Human–Companion Animal Relationship Duration, Companion Animal Life Stage, and Relationship Quality

**DOI:** 10.3390/ani14111606

**Published:** 2024-05-29

**Authors:** Annalyse Ellis, Steve Loughnan, Roxanne D. Hawkins, Sarah C. E. Stanton

**Affiliations:** 1 Department of Psychology, School of Philosophy, Psychology and Language Sciences, University of Edinburgh, Edinburgh EH8 9AD, UK; steve.loughnan@ed.ac.uk (S.L.); sarah.stanton@ed.ac.uk (S.C.E.S.); 2 Department of Clinical and Health Psychology, School of Health in Social Science, University of Edinburgh, Edinburgh EH8 9AG, UK; roxanne.hawkins@ed.ac.uk

**Keywords:** human–animal interaction, companion animal, perceived pet responsiveness, self-expansion, pet ownership, attachment bond

## Abstract

**Simple Summary:**

The present research explored the links between human–pet relationship duration, pet life stage, and four markers of relationship quality: pet-related self-expansion, perceived pet responsiveness, perceived pet insensitivity, and human–animal bond, for dog and cat owners. Human–pet relationship duration negatively predicted self-expansion for dog and cat owners, and self-expansion was also higher for owners of younger pets compared to owners of older pets. Perceived pet responsiveness, perceived pet insensitivity, and human–animal bond were not associated with relationship duration or pet life stage. The findings of the present research indicate that human–pet relationship duration and pet life stage have implications for how much people feel a pet helps them grow.

**Abstract:**

Although many companion animal (or “pet”) owners report that their relationships with their pets are important, we know little about how animal ownership duration and animal life stage are related to relationship quality. In a sample of 1303 dog and cat owners, the present research explored the associations between relationship duration, pet life stage (puppy/kitten, young adult, mature adult, and senior), and four markers of relationship quality: pet-related self-expansion, perceived pet responsiveness, perceived pet insensitivity, and human–animal bond. We found that relationship duration was negatively and linearly associated with self-expansion for both dog and cat owners. Results comparing relationship quality markers across pet life stages revealed that for both dog and cat owners, self-expansion was higher for owners of younger animals compared to owners of older animals. There were no significant effects for perceived pet responsiveness, perceived pet insensitivity, or human–animal bond based on relationship duration or animal life stage. These findings indicate that the duration of the relationship with one’s pet and animal life stage have implications for how much people feel a pet helps them grow, whereas other markers of human–pet relationship quality likely vary based on other experiences.

## 1. Introduction

Many people report that their relationship with their companion animals (hereafter, for brevity, “pets”) is highly important [1,2]. For most, acquiring a pet is a serious commitment and signals the start of a long-term relationship. With cats typically living for around 15 years and dogs typically living for 10–15 years, many people will enjoy a relationship with their pet that spans a decade [3]. Furthermore, because cats and dogs have faster life cycles than humans, owners may well witness their pets experience multiple life stages (e.g., from puppy or kitten to senior) [4,5]. Dogs’ and cats’ behaviors and needs change throughout their different life stages, which could impact the nature of the human–pet relationship [4,5,6,7]. Despite these relationships being important, long-lasting, and potentially changing, relatively little is known regarding human–pet relationships compared to human–human relationships. This disparity is especially apparent in the understanding of how markers of relationship quality are associated with both relationship duration and pets’ shifting life stages. The current study investigated the associations between human–pet relationship duration, pet life stage, and four markers of relationship quality: pet-related self-expansion, perceived pet responsiveness, perceived pet insensitivity, and human–animal bond, with the goal of increasing our understanding of the nuances of the human–pet relationship.

### 1.1. Self-Expansion

Self-expansion is the process of adding new positive content to the self, often through close relationships and exciting new experiences [8,9]. There are two main aspects of the self-expansion model: (1) humans are motivated to self-expand in order to increase self-efficacy and reach their goals and (2) they do so typically through developing and maintaining close relationships with others and integrating close others into their sense of self [9]. In human–human relationships, self-expansion can contribute to greater feelings of self-efficacy [10], greater positive self-concept [11,12], and better mental well-being [13,14]. Self-expansion also contributes to better relationship quality and more relationship satisfaction [15]. Several studies in the human–human relationship field have suggested that self-expansion can change over time. For example, Mattingly et al. [16] found that self-expansion increased slightly over a 9-month period for participants who had been in a romantic relationship for less than 6 months. It is theorized that self-expansion increases rapidly at the beginning of relationships and then decreases over time [8]. This trend is argued to occur because there are many opportunities for engaging in novel activities, learning more about a close other, and integrating the new close other’s resources and perspectives into the sense of self within the context of the new relationship. However, as relationships continue over time, these natural opportunities for self-expansion wane [8].

Within human–pet relationships, research on the role of self-expansion is limited, with only one prior study focusing on self-expansion in a similar way to how we understand it in human–human relationships [17]. This study found that self-expansion in the human–pet relationship is higher in dog owners than in cat owners and that self-expansion is associated with higher levels of positive affect and lower levels of loneliness. However, no studies to this point have explored the association between human–pet relationship duration and self-expansion. Based on human–human research, we would expect that self-expansion is negatively associated with relationship duration in human–pet relationships [8]. Furthermore, relationships with a new animal, particularly a puppy or kitten, may offer opportunities for self-expanding activities that would perhaps wane over time as the pet aged.

### 1.2. Perceived Responsiveness and Insensitivity

Perceived responsiveness and insensitivity have been studied extensively within human–human relationships (particularly within romantic relationships) and, to a lesser extent, within human–pet relationships. Perceived responsiveness is the extent to which individuals believe a close other to be validating, caring, and understanding, while perceived insensitivity is the inverse of this [18,19]. Perceived responsiveness has implications for health and well-being, with perceived responsiveness being associated with lower depression, anxiety, and bodily stress [20,21,22]. Although there is limited information related to whether perceived responsiveness and insensitivity vary based on relationship duration, theoretically, it could be expected that these markers of relationship quality would change throughout the duration of the relationship based on factors such as “cycles of responsiveness”, in which individuals in a relationship are alternatingly and repeatedly responsive to one another [23], and “stress spillover”, in which external stressors are associated with more negative relational interactions [24,25]. Furthermore, research on married couples demonstrates that marital happiness declines normatively over time [26], potentially suggesting that perceived responsiveness (which contributes to marital happiness) may show a similar pattern.

As with pet-focused self-expansion, there has only been one study of perceived pet responsiveness and insensitivity [17]. This study found that dog owners perceive their pets to be significantly more responsive and less insensitive than cat owners. Perceived pet responsiveness did not predict any mental well-being outcomes in this study; however, perceived pet insensitivity predicted more depression and anxiety symptoms, negative affect, and loneliness. However, this study did not explore the associations between perceived pet responsiveness and insensitivity, relationship duration, or pet life stage.

### 1.3. Human–Animal Bond

Unlike pet-related self-expansion and perceived pet responsiveness and insensitivity, human–animal attachment bond (hereafter, for brevity, “human–animal bond”) has been long studied within human–pet relationships. An attachment bond is a relationship that provides both comfort and security [27], and the human–animal bond specifically is defined as a mutually beneficial relationship between a human and a pet that contributes to the well-being of both [28]. Most pet owners report a close and supportive relationship with their pets [29,30], and several studies have found that the human–pet bond tends to be very strong [31,32]. Pet ownership provides a unique opportunity for the formation of a bond due to pet availability, pets’ attunement to their owners’ emotions and needs, and their ability to provide comfort [33]. Human–animal bonds also have implications for mental well-being [34,35].

There is limited past research about when a bond forms between humans and their pets, how that bond may vary based on relationship duration, and if bonds differ based on pet life stage. One longitudinal study found that children surveyed over a five-year period showed stable bonds with their pets [36], while another found that adolescents between the ages of 11 and 15 experience decreases in their bonds with their pets over the course of those four years [37]. However, these studies focus on children and adolescents specifically, whereas the present research focuses on adults. Furthermore, although there is no research related to pet life stage and human–animal bond, there is evidence that fewer mental well-being benefits are derived from the relationship when the animals are puppies or kittens [38] or when pets are older due to issues such as anticipatory grief associated with pets’ aging or illness [39,40,41], indicating that perhaps the human–animal bond is weaker at these stages.

### 1.4. Research Aim

People have long-lasting relationships with their pets, relationships which frequently span multiple stages of the animal’s life. Despite prior work on human–human relationships showing the dynamic nature of these links, this has been typically neglected when examining human–pet relationships. The current study aimed to examine whether human–pet relationship duration and pet life stage are associated with self-expansion, perceived pet responsiveness and insensitivity, and human–animal bond. Dogs and cats were examined separately because of their different life expectancies and to explore the differences in how these relationship quality markers differ depending on pet type. We did not make any a priori hypotheses, treating the current work as exploratory.

## 2. Materials and Methods

### 2.1. Participants

A total of 1455 participants were recruited between June 2022 and February 2023. On examination, 120 of these participants were excluded due to incomplete data or failing attention checks. A further 5 participants reported pet ages that were impossible based on dog and cat life expectancies and were therefore excluded due to data unreliability, and 6 participants were excluded for being under 18 years of age, as this study focused only on adults. Finally, 21 pet owners who reported having had their pet for more than 15 years were excluded due to small participant numbers after this point (1.6%), leaving a total of 1303 included participants. See Table 1 for demographic information.

### 2.2. Materials and Measures

Data were taken from a larger study of human–pet relationships. All study measures can be found at https://osf.io/9vdgu/. The current study used a version of the Companion Animals Self-Expansion Scale (CASES) [42], the Perceived Pet Responsiveness Scale (PPRS) [17], the Perceived Pet Insensitivity Scale (PPIS) [17], and the Lexington Attachment to Pets Scale (LAPS) [43], as well as several demographic questions related to age, income, gender, pet age and breed, and year that the pet was acquired. 

The CASES [42] is a 15-item questionnaire developed to measure self-expansion within the human–pet relationship. The scale utilized in this study was a 14-item version of the full CASES. Participants were asked to respond to each question (e.g., “How much does having a pet result in your having new experiences?”; “How much does your pet provide a source of exciting experiences?”) on a 7-point scale (1 = not very much, 7 = very much). Cronbach’s alpha for the sample was 0.96. 

The PPRS [17] is a 24-item questionnaire developed to measure perceived responsiveness within the human–pet relationship. Participants were asked to indicate their level of agreement with each statement (e.g., “My pet really listens to me”; “My pet knows me well”) on a 7-point scale (1 = strongly disagree, 7 = strongly agree). Cronbach’s alpha for the sample was 0.97.

The PPIS [17] is a 24-item questionnaire developed to measure perceived pet insensitivity within the human–pet relationship. Participants were asked to indicate their level of agreement with each statement (e.g., “My pet does not accept my feelings and concerns”; “My pet is NOT attentive to my needs”) on a 7-point scale (1 = strongly disagree, 7 = strongly agree). Cronbach’s alpha for the sample was 0.97.

The LAPS [43] is a 23-item questionnaire designed to measure the human–animal bond. Participants were asked to indicate their level of agreement with each statement (e.g., “My pet means more to me than any of my friends”) on a 4-point scale (1 = strongly disagree, 4 = strongly agree). Cronbach’s alpha for the sample was 0.94.

### 2.3. Procedure

Participants were recruited from Prolific Academic and compensated GBP 1.30 for their participation. Participants were eligible to participate in the study if they were (1) 18 years of age or older, (2) living in the UK, and (3) owning a pet cat or dog. Upon consenting to participate in the study, participants were asked to complete demographic questions, followed by measures of pet-related self-expansion, perceived pet responsiveness, perceived pet insensitivity, and human–animal bond. Attention check questions were randomized throughout the survey. Scales were randomized within the survey, as were the items in each scale.

## 3. Results

### 3.1. Descriptive Statistics and Correlations

Descriptive statistics and correlations are displayed in Table 2. All relational variables displayed significant correlations with one another. For both dog and cat owners, self-expansion correlated negatively with pet age and relationship duration. Perceived pet responsiveness did not correlate significantly with pet age for either dog or cat owners, but did correlate negatively with relationship duration for cat owners. Neither perceived pet insensitivity nor human–animal bond displayed a significant relationship with pet age or relationship duration for either dog or cat owners.

### 3.2. Regressions: Dogs

Multilinear regressions were conducted with dog-owning participants to explore the relationship between human–dog relationship duration and relational variables while controlling for demographic variables. Prior to completing the regression analysis, age and relationship duration were grand mean-centered. Additionally, participants identifying as non-binary or not reporting a gender were excluded due to the small number (*n* = 15) of these participants. Gender was coded for analyses (1 = men, 0 = women). Four multilinear regressions were conducted (one for each relationship variable), controlling for pet owner age and pet owner gender, with relationship duration as a predictor (We initially conducted multilinear regressions that included both linear and quadratic effects of relationship duration in order to explore both linear and non-linear associations between relationship duration and the outcome variables; however, the quadratic effects of relationship duration were not significant for any model).

Table 3 reports the results of the four models in which self-expansion, perceived pet responsiveness, perceived pet insensitivity, and human–animal bond were outcome variables. The linear effects of relationship duration were explored in these models as a predictor of each of these relational variables. Relationship duration was a significant negative predictor and age was a significant positive predictor only for the model in which self-expansion was the outcome. Gender was a significant negative predictor of self-expansion, perceived pet responsiveness, and human–animal bond and a significant positive predictor of perceived pet insensitivity.

### 3.3. Regressions: Cats

Multilinear regressions were conducted with cat-owning participants to explore the relationship between human–cat relationship duration and relational variables while controlling for demographic variables. The analysis was completed following the same protocol as described for dog-owning participants above. 

Table 4 reports the results of the four models in which self-expansion, perceived pet responsiveness, perceived pet insensitivity, and human–animal bond were outcome variables. The linear effects of relationship duration were explored in these models as a predictor of each of these relational variables. Relationship duration was a significant negative predictor only for the model in which self-expansion was the outcome, gender was a significant negative predictor of perceived pet responsiveness and human–animal bond, and age was a significant positive predictor of perceived pet insensitivity and a significant negative predictor of human–animal bond.

### 3.4. Visualizations of Human–Pet Relationship Duration and Relational Variables

**Figure 1:**
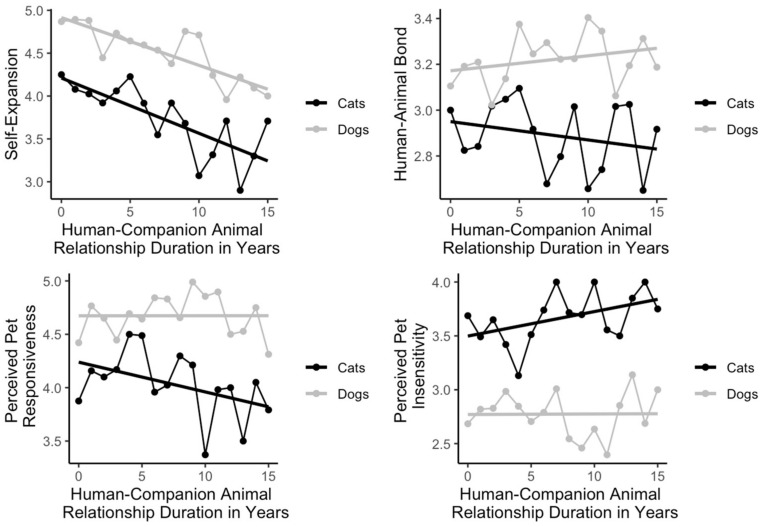
Visualization of the association between relationship duration and relational variables.

The associations between all relational variables and relationship duration for dogs and cats are visualized in Figure 1. Self-expansion showed the strongest pattern of decrease associated with relationship duration, whereas human–animal bond, perceived pet responsiveness, and perceived pet insensitivity displayed much more modest patterns of relationship with relationship duration.

### 3.5. Pet Type and Life Stage

Pets were sorted into life stage groups based on American Animal Hospital Association guidelines [4,5]. Life stages for dogs were defined as puppy (*n* = 35), young adult (*n* = 216), mature adult (*n* = 188), and senior (*n* = 257). Some dogs (*n* = 47) were unable to be sorted into life stage groups due to a lack of breed information or mixed breed status and were therefore excluded from this section of the analysis. Life stages for cats were defined as kitten (*n* = 42), young adult (*n* = 201), mature adult (*n* = 167), and senior (*n* = 150). 

One-way ANOVAs were conducted to explore the association between dog life stage and self-expansion, perceived pet responsiveness, perceived pet insensitivity, and human–animal bond. The analysis revealed a significant difference in self-expansion for dog owners by life stage groups (*F*(3, 692) = 5.20, *p* = 0.001, *ηp*^2^ = 0.02). Post-hoc analysis revealed that the mean value of self-expansion was significantly higher for owners of puppies than for owners of mature adult dogs (*t*(44.76) = 2.04, *p* = 0.047), significantly higher for owners of puppies than for owners of senior dogs (*t*(43.72) = 3.27, *p* = 0.002), significantly higher for owners of young adult dogs than for owners of senior dogs (*t*(451.66) = 2.54, *p* = 0.011), and significantly higher for owners of mature adult dogs than for owners of senior dogs (*t*(424.46) = 2.44, *p* = 0.015). One-way ANOVAs revealed no significant difference in perceived pet responsiveness (*F*(3, 692) = 0.44, *p* = 0.725, *ηp*^2^ = 0.002), perceived pet insensitivity (*F*(3, 692) = 0.87, *p* = 0.461, *ηp*^2^ = 0.004), or human–animal bond (*F*(3, 692) = 2.03, *p* = 0.109, *ηp*^2^ = 0.009) between any of the dog life stage groups. 

**Figure 2:**
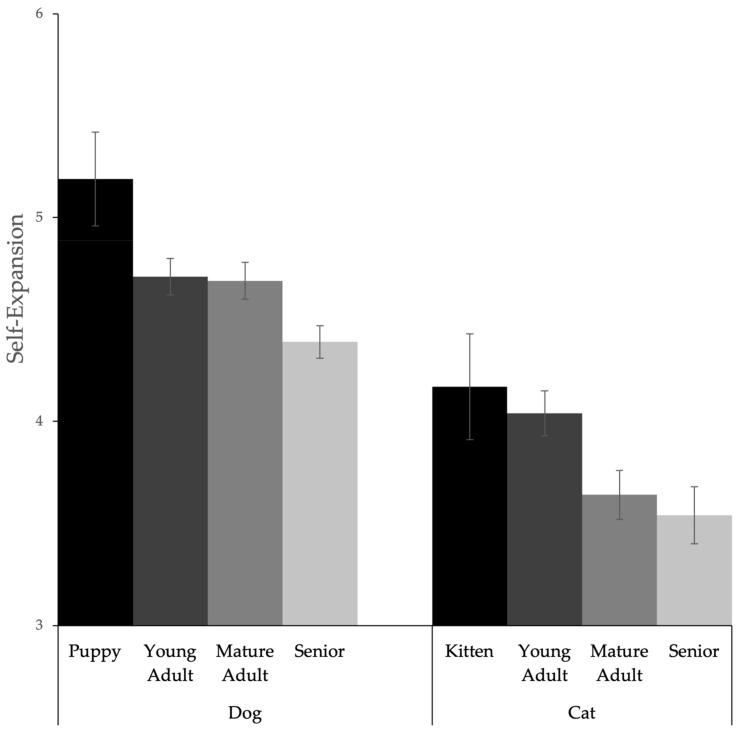
Self-expansion by life stage and type of pet.

Similarly, one-way ANOVAs were conducted to explore the association between cat life stage and self-expansion, perceived pet responsiveness, perceived pet insensitivity, and human–animal bond. The analysis revealed a significant difference in self-expansion for cat owners by life stage groups (*F*(3, 556) = 4.09, *p* = 0.007, *ηp*^2^ = 0.02). Post-hoc analysis revealed that the mean value of self-expansion was significantly higher for owners of kittens than for owners of senior cats (*t*(65.27) = 2.12, *p* = 0.038), higher for owners of young adult cats than for owners of mature adult cats (*t*(348.43) = 2.45, *p* = 0.015), and higher for owners of young adult cats than for owners of senior cats (*t*(305.99) = 2.85, *p* = 0.005). The association between self-expansion and pet life stage for both cats and dogs is displayed in Figure 2. One-way ANOVAs revealed no significant difference in perceived pet responsiveness (*F*(3, 556) = 1.20, *p* = 0.311, *ηp*^2^ = 0.006), perceived pet insensitivity (*F*(3, 556) = 0.72, *p* = 0.539, *ηp*^2^ = 0.004), or human–animal bond (*F*(3, 556) = 2.29, *p* = 0.077, *ηp*^2^ = 0.01) between any of the cat life stage groups. Figure 2 displays self-expansion by life stage and type of pet. Table 5 reports the means and standard deviations for all relational variables for each life stage of both dogs and cats.

## 4. Discussion

Although many pet owners report that their relationships with their pets are highly important [1,2], comparatively little research has been conducted related to markers of relationship quality within these relationships as opposed to human–human relationships. This study sought to explore if pet-related self-expansion, perceived pet responsiveness, perceived pet insensitivity, and human–animal bond differ based on the duration of the relationship and pet life stage. Self-expansion was negatively predicted by relationship duration for both cat and dog owners, and also emerged as the only marker of relationship quality that displayed significant differences related to pet life stage for both cat and dog owners. Human–animal bond, perceived pet responsiveness, and perceived pet insensitivity had no significant associations with either relationship duration or pet life stage.

### 4.1. Self-Expansion

The results related to self-expansion and relationship duration are aligned with the limited existing research on self-expansion within human–human relationships and human–pet relationships. The literature related to self-expansion in human–human relationships theorizes that levels of self-expansion are often high at the beginning of relationships, typically declining over time as new experiences within the relationship decrease [8]. A similar effect was found in this study of human–animal relationships: regressions indicated that relationship duration negatively predicted self-expansion. Perhaps, similarly to human–human relationships, there are decreases in novel activities and opportunities for growth and learning in human–pet relationships over time as owners and pets settle into routines. For example, when an individual acquires a new pet, they may have the opportunity to go to new places with their animal, learn about their animal’s habits and personality, meet new people because of their animal, and engage in other new activities with their animal (e.g., training). After having had their pet for a longer period of time, these activities may no longer be new or exciting, accounting for the downward trend in self-expansion related to relationship duration. Additionally, identity as a pet owner may play a role in this association; when a new pet owner acquires their animal, they may integrate the identity of “pet owner” into their sense of self, but, once that new identity is integrated, the pet owner will not continue to self-expand related to that identity.

Analysis related to self-expansion and pet life stage revealed that self-expansion was highest for owners of puppies and kittens and lowest for owners of senior animals. Owning a puppy or kitten may provide the most opportunities for self-expansion: not only are these relationships likely to be new and novel, but puppies and kittens often require the highest amount of interaction, such as care and play, creating accessible opportunities for self-expansion. These findings, as well as the findings regarding the negative association between relationship duration and self-expansion, imply that human–pet relationships require maintenance, just as human–human relationships do. For example, in human–human relationships, engaging in novel and exciting activities can be a common relationship maintenance strategy and an opportunity for increasing the experience of self-expansion [44,45,46]. Within human–pet relationships, pet owners may need to seek ways to inject self-expansion into their daily lives, especially as their pets age or as the relationship duration increases. Opportunities for self-expansion can include simple activities such as walking one’s dog to a new place or playing with a new toy with one’s cat. This may be important when considering well-being outcomes as perhaps owners who make efforts to maintain self-expanding activities and interactions with their pets will have better well-being outcomes. However, these findings should be interpreted with caution as pet life stage, while significant, only accounted for 2% of the variation in self-expansion.

### 4.2. Perceived Responsiveness and Insensitivity

Neither perceived pet responsiveness nor insensitivity displayed a significant relationship with relationship duration or pet life stage. It is possible that there are periods in which each individual pet owner may feel that their pet is more or less responsive or insensitive, which could explain the lack of a pattern seen in the present work. Previous research on the subject has found that cycles of responsiveness can occur in relationships in which individuals in a relationship dyad create a repeating sequence of being responsive to one another [23]. Although this example has only been studied within human–human relationships, it is possible that cycles of responsiveness may be present in human–pet relationships as well. For example, pet owners may be responsive to their pets’ needs by providing enrichment and affection, and pets may respond in kind by spending time with their owner and engaging positively, promoting further owner engagement and responsiveness, thus creating a cycle of responsiveness. Moreover, previous research has also found that stress spillover is associated with more negative relational interactions [24,25]. In the human–pet relationship, this may present as the pet owner not having the time or energy to provide enrichment, affection, or time to their pet, resulting in negative behaviors from the pet and negative interactions between the owner and the animal. Both of these examples indicate that perhaps perceived pet responsiveness and perceived pet insensitivity are more associated with day-to-day interactions rather than displaying a long-term pattern. Additionally, perceived pet responsiveness and perceived pet insensitivity may be moderated by other variables, such as anthropomorphism (attributing human characteristics to a pet) or individual differences between pet owners (e.g., personality). 

### 4.3. Human–Animal Bond

There was no significant association between relationship duration and human–animal bond for either cat or dog owners. The existing literature indicates that adult owners of pets are, overall, strongly bonded with their pets, and the participants in this study demonstrated a strong bond with their pets as well [31,32]. Although past studies did not control for relationship duration or pet life stage, this study indicates that pet owners’ bond with their pets remains relatively constant regardless of these two variables. This is surprising considering the literature that indicates that the infantile features often seen in puppies and kittens promote caregiving behavior and bonding for pet owners [47,48,49], indicating that perhaps the human–animal bond would be strongest for owners of puppies or kittens, a theory not supported by the current findings. Furthermore, the existing literature indicates that some pet owners experience potential psychological distancing due to concerns such as anticipatory grief associated with pets’ aging or illness [39,40,41]. It could therefore be expected that the human–pet bond would decrease over time as an animal ages, but this was also not supported by our findings. 

### 4.4. Implications for Human Well-Being

The present study has implications for the mental well-being of pet owners. The well-being benefits of human–pet relationships have been well-researched, but have yielded varying results, with some studies indicating positive well-being outcomes [50,51,52,53], some reporting neutral well-being outcomes [51,54,55,56,57], and others reporting negative well-being outcomes [58,59]. Although perceived pet responsiveness, perceived pet insensitivity, and human–animal bond did not display a significant relationship with pet relationship duration or pet life stage, self-expansion displayed a significant association with relationship duration and pet life stage for both dog and cat owners. Past research has shown that self-expansion is associated with more positive affect and less loneliness [17]. The present study indicates that perhaps mental well-being may be associated with relationship duration and pet life stage because of changes in this relational variable.

### 4.5. Implications for Pet Relinquishment and Welfare

The present research also has implications for pet relinquishment and welfare. Relinquishment is commonplace, responsible for around 25% of shelter intakes in the United States in 2023 [60]. Furthermore, in the UK, 18,779 dogs and 33,744 cats came into the care of the RSPCA during their 2022–2023 fiscal year [61]. Common causes of relinquishment include having too many animals, poor human health, no longer wanting the animal, housing issues, animal behavioral issues, financial issues, life changes, poor animal health, undesirable animal characteristics, and personal issues [62,63]. Peak relinquishments occur when cats and dogs are between one and three years of age, and this aligns with the young adult life stage for most dogs and all cats [64]. Interestingly, this is also the life stage at which both dog and cat owners’ self-expansion begins to drop. This could indicate that relinquishment is partially driven by declining self-expansion, and preserving self-expansion might help maintain the quality of the human–pet relationship.

### 4.6. Limitations

A few limitations of this study should be noted. Relationship duration data were collected based on years of ownership rather than months. Although this likely did not impact the analysis for owners who had their pets for longer periods of time, this did limit our analysis for owners who had had their pets for shorter periods of time (e.g., less than two years). Having data related to relationship duration in smaller time increments will be useful for capturing the nuanced differences during this critical period. Additionally, we did not control for the age of the pet at the time of acquisition, and this could potentially play a role in participants’ experience of the markers of relationship quality explored in the current research. While this study purposefully only recruited dog and cat owners as these are the two most common pet types, we are unable to speak to how these processes might differ for other types of pets. Finally, a key limitation of this study is its cross-sectional design. This study is not able to provide information on how these relationships change over time, only how they look at a single time point.

### 4.7. Future Directions

This study opens the door to several avenues of future research. More research is needed to examine these relational variables (particularly self-expansion, perceived pet responsiveness, and perceived pet insensitivity) within the human–pet context, as well as across different pet types. Second, longitudinal research is needed, especially beginning in the earliest stages of the human–pet relationship, exploring possible relationship changes throughout the duration of a pet’s life. This type of research could provide important insight into relational dynamics such as the variables explored in these studies. Additionally, while this study focused on individuals living in the United Kingdom, it would be interesting to replicate this research with populations in other countries and cultures. Furthermore, longitudinal research would provide better insight into the formation and quality of the human–animal bond.

## 5. Conclusions

The present research sought to better understand the associations between self-expansion, perceived pet responsiveness and insensitivity, human–animal bond, and relationship duration and pet life stage. Our findings indicate that some markers of relationship quality vary based on the duration of the relationship, as well as within the context of pet life stage. These results provide further insight into the complexities of these relationships and have implications for our understanding of how these relationships may develop and change. This study also has implications for pet and owner well-being, especially related to pet relinquishment. The present work points to the critical and previously neglected importance of understanding both relationship duration and pet life stage for pet–owner relationship quality.

## Figures and Tables

**Table 1:** Participant demographics.

	*N* = 1303
Gender	
Women	705 (54.1%)
Men	583 (44.7%)
Non-Binary	14 (1.1%)
Prefer not to say	1 (0.1%)
Participant Age	
Mean (SD)	40.9 (13.8)
Median [Min, Max]	39.0 [18, 80]
Pet Type	
Cat	560 (43.0%)
Dog	743 (57.0%)
Pet Age	
Mean (SD)	6.9 (4.2)
Median [Min, Max]	7.0 [0, 24]

**Table 2:** Descriptive statistics and correlations among study variables. Top diagonal (dark numbers) is dogs, bottom diagonal (light numbers) is cats.

	M (Dogs)	SD (Dogs)	M (Cats)	SD (Cats)	Pet Age	Relationship Duration	Self- Expansion	Responsiveness	Insensitivity	Bond
Pet Age	6.34	3.97	7.53	4.29	-	0.892 ***	−0.140 ***	0.020	−0.039	0.070
Relationship Duration	5.71	3.85	6.24	3.96	0.784 ***	-	−0.141 ***	0.027	−0.037	0.061
Self-Expansion	4.62	1.33	3.80	1.62	−0.118 **	−0.164 ***	-	0.459 ***	−0.441 ***	0.412 ***
Responsiveness	4.71	1.44	4.08	1.59	−0.049	−0.085 *	0.502 ***	-	−0.603 ***	0.526 ***
Insensitivity	2.78	1.42	3.63	1.59	0.022	0.066	−0.447 ***	−0.643 ***	-	−0.418 ***
Bond	3.22	0.66	2.89	0.78	−0.003	−0.028	0.442 ***	0.587 ***	−0.511 ***	-

Note: * *p* < 0.05, ** *p* < 0.01, *** *p* < 0.001.

**Table 3:** Results of multilinear regressions for self-expansion, responsiveness, insensitivity, and human–animal bond for dog owners.

	Self-Expansion β [95% CI]	Responsiveness β [95% CI]	Insensitivity β [95% CI]	Human–Animal Bond β [95% CI]
(Intercept)	4.775 *** [4.646, 4.904]	4.922 *** [4.782, 5.062]	2.659 *** [2.519, 2.799]	3.280 *** [3.215, 3.345]
Pet Owner Age	0.007 * [0.001, 0.014]	−0.0005 [−0.008, 0.007]	0.004 [−0.004, 0.011]	−0.002 [−0.005, 0.002]
Gender	−0.355 ** [−0.545, −0.165]	−0.484 ** [−0.691, −0.277]	0.268 * [0.062, 0.474]	−0.141 ** [−0.236, −0.045]
Relationship Duration	−0.055 ** [−0.079, −0.030]	0.007 [−0.019, 0.034]	−0.012 [−0.039, 0.015]	0.010 [−0.002, 0.023]
R^2^/R^2^ Adjusted	0.046/0.042	0.029/0.025	0.012/0.007	0.015/0.011

Note: * *p* < 0.05, ** *p* < 0.01, *** *p* < 0.001.

**Table 4:** Results of multilinear regressions for self-expansion, responsiveness, insensitivity, and human–animal bond for cat owners.

	Self-Expansion β [95% CI]	Responsiveness β [95% CI]	Insensitivity β [95% CI]	Human–Animal Bond β [95% CI]
(Intercept)	3.849 *** [3.669, 4.029]	4.299 *** [4.124, 4.475]	3.523 *** [3.347, 3.700]	2.973 *** [2.886, 3.059]
Human Age	−0.009 [−0.019, 0.001]	−0.006 [−0.016, 0.004]	0.016 ** [0.006, 0.026]	−0.006 * [−0.011, −0.001]
Gender	−0.120 [−0.390, 0.151]	−0.484 ** [−0.749, −0.220]	0.252 [−0.013, 0.518]	−0.187 ** [−0.318, −0.057]
Relationship Duration	−0.063 ** [−0.097, −0.029]	−0.028 [−0.062, 0.005]	0.016 [−0.018, 0.050]	−0.002 [−0.018, 0.015]
R^2^/R^2^ Adjusted	0.034/0.029	0.033/0.027	0.026/0.021	0.026/0.021

Note: * *p* < 0.05, ** *p* < 0.01, *** *p* < 0.001.

**Table 5:** Means and standard deviations of each life stage group for dog and cat owners.

	Self-Expansion		Perceived Pet Responsiveness		Perceived Pet Insensitivity		Human–Animal Bond	
	Mean	SD	Mean	SD	Mean	SD	Mean	SD
Puppies	5.19	1.35	4.71	1.34	2.79	1.43	3.17	0.69
Young adult dogs	4.71	1.39	4.63	1.50	2.89	1.56	3.13	0.70
Mature adult dogs	4.69	1.21	4.79	1.32	2.78	1.38	3.27	0.63
Senior dogs	4.39	1.35	4.75	1.47	2.67	1.32	3.25	0.65
Kittens	4.17	1.69	4.04	1.66	3.56	1.69	2.77	0.67
Young adult cats	4.04	1.54	4.25	1.55	3.52	1.53	2.99	0.76
Mature adult cats	3.64	1.60	3.95	1.65	3.76	1.65	2.79	0.85
Senior cats	3.54	1.68	4.03	1.55	3.64	1.59	2.91	0.75

## Data Availability

The data presented in this study are available upon request from the corresponding author. The data are not publicly available due to privacy reasons. Materials and codes are available on the Open Science Framework at https://osf.io/9vdgu/.
